# Machine learning-augmented biomarkers in mid-pregnancy Down syndrome screening improve prediction of small-for-gestational-age infants

**DOI:** 10.1186/s13023-025-04027-1

**Published:** 2025-10-01

**Authors:** Bin Zhang, Xusheng Chen, Zhaolong Zhan, Sijie Xi, Yinglu Zhang, He Dong, Xiaosong Yuan

**Affiliations:** Department of Medical Genetics, Changzhou Maternal and Child Health Care Hospital, Changzhou Medical Center, Nanjing Medical University, Changzhou, China

**Keywords:** Unconjugated estriol, Adverse fetal growth outcomes, Machine learning, Small for gestational age infants, Prenatal screening, Prediction models

## Abstract

**Background:**

Adverse fetal growth outcomes (AFGO), primarily characterized by small-for-gestational age (SGA), large-for-gestational age (LGA), low birth weight (LBW) neonates, and macrosomia (Mac), present substantial challenges in early prediction. This study aims to 1) establish a predictive probability for AFGO using routine biochemical markers from prenatal Down syndrome screening, and 2) evaluate the performance of machine learning-based prediction models that incorporate these biomarkers and maternal characteristics for AFGO identification.

**Methods:**

A retrospective analysis was conducted on 2533 singleton deliveries from 2015 to 2017, with available data on early second-trimester biomarkers [α-fetoprotein (AFP), free β-human chorionic gonadotropin (fβ-hCG), and unconjugated estriol (uE3)], as well as pregnancy outcomes.

**Results:**

Serum uE3 demonstrated higher predictive performance for AFGO compared to fβ-hCG or AFP alone, with higher area under the curve (AUC) values in receiver operating characteristic (ROC) analyses (SGA: 0.626 vs. 0.501/0.500; LGA: 0.557 vs. 0.502/0.537; LBW: 0.614 vs. 0.543/0.559; Mac: 0.546 vs. 0.532/0.519). To improve AFGO prediction, we developed four machine learning-based models. Gradient boosting machine (GBM) and generalized linear model (GLM) models demonstrated optimal performance for SGA prediction, achieving AUC values of 0.873 and 0.706, respectively, in the training set (n = 1782, SGA 143), and 0.717 and 0.739 in the test set (n = 751, SGA 68).

**Conclusion:**

Serum uE3 is superior to fβ-hCG and AFP in predicting AFGO. GBM and GLM models significantly enhance SGA prediction performance, highlighting the potential of integrating routine prenatal screening biomarkers with machine learning for early identification of AFGO.

**Supplementary Information:**

The online version contains supplementary material available at 10.1186/s13023-025-04027-1.

## Introduction

Birth weight serves as a critical indicator for assessing fetal health status and intrauterine growth conditions. Two prevalent adverse fetal growth outcomes (AFGO) are small-for-gestational-age (SGA) and large-for-gestational-age (LGA), which refer to babies whose birth weights fall below the 10th percentile and above the 90th percentile for their gestational age, respectively [1]. These conditions pose significant risks to maternal and neonatal health and are of particular concern due to their association with long-term metabolic sequelae in offspring, including insulin resistance, obesity, and cardiovascular disorders later in life [2, 3]. Consequently, the early detection of AFGO is a pivotal objective in contemporary prenatal care. Timely identification allows for targeted monitoring and interventions, thereby mitigating adverse outcomes for both mothers and infants.

Routine prenatal screening programs, such as mid-trimester Down syndrome screening, offer a valuable opportunity for the early detection of potential growth issues. These screenings typically collect maternal serum biomarkers including alpha-fetoprotein (AFP), free β-human chorionic gonadotropin (fβ-hCG), and unconjugated estriol (uE3). Beyond their established role in fetal aneuploidy detection and neural tube defects, emerging evidence suggests that deviations in these biomarkers may also reflect placental dysfunction and impaired fetal growth [4]. For instance, elevated AFP levels in the second trimester have been associated with placental vascular anomalies and adverse pregnancy outcomes, including fetal growth restriction (FGR) and low birth weight (LBW) [5]. Similarly, abnormal uE3 and fβ-hCG profiles correlate with trophoblast insufficiency and disrupted steroidogenesis, which may precede suboptimal fetal development [6, 7]. Despite these pathophysiological insights, current clinical practice rarely integrates mid-trimester serum markers into systematic risk assessment for fetal growth abnormalities, relying instead on ultrasound-based biometric measurements and maternal comorbidities, which exhibit limited sensitivity in early identification [8].

Machine learning (ML), a pivotal subfield of artificial intelligence, has emerged as a transformative methodology for developing predictive tools in perinatal care [9–12]. By integrating heterogeneous data streams, including maternal demographics, obstetric histories, and multi-omics biomarkers, ML algorithms demonstrate superior pattern recognition capabilities in stratifying pregnancy risks compared to conventional statistical approaches [13]. Growing evidence underscores the clinical value of ML in predicting adverse perinatal outcomes and pregnancy complications such as stillbirth, shoulder dystocia, preterm birth (PTB), birth weight, FGR, SGA, macrosomia (Mac), gestational diabetes mellitus (GDM), preeclampsia (PE), and hypertensive disorders of pregnancy [14–27]. This study aimed to leverage routinely collected biochemical markers from mid-pregnancy prenatal screening for Down syndrome to develop predictive models for AFGO. For instance, aberrant fetal growth patterns are often associated with rare genetic syndromes (e.g., Russell-Silver syndrome, Temple syndrome), placental mosaicism, or metabolic disorders (e.g., Smith-Lemli-Opitz syndrome). Early prediction of SGA using these widely available biomarkers could thus facilitate the identification of rare diseases that currently lack systematic screening protocols during prenatal care. Additionally, we developed and evaluated ML-based prediction models that incorporate these biomarkers alongside maternal characteristics within prenatal screening.

## Materials and methods

### Participants and data collection

This observational cohort study initially enrolled a total of 2634 consecutive women with singleton pregnancies who underwent routine serum screening for Down's syndrome and subsequently delivered at Changzhou Maternity and Child Health Care (MCHC) Hospital between October 2015 and March 2017. Participants were included in the study if they met the following criteria: (1) pregnancy stage between 15 and 20 weeks of gestation with negative screening results; (2) availability of clear and integrated records; and (3) gave birth to a live neonate without any congenital defects. Exclusion criteria included: (1) presence of pre-pregnancy comorbidities, including chronic renal, hepatic, or cardiac diseases; hypertension; diabetes mellitus; thyroid disorders; immune rheumatic diseases; and syphilis; and (2) engagement in adverse health behaviors during the current pregnancy. To isolate the independent predictive value of routine biochemical markers (AFP, uE3, and fβ-hCG) for AFGO, we restricted the cohort to healthy, screen-negative (low-risk) pregnancies. Since risk calculations for aneuploidies and neural tube defects were derived from these same markers, high-risk screening results (indicating abnormal marker levels) were excluded to minimize confounding. Maternal comorbidities and congenital anomalies were also excluded to focus on the markers’ contribution in a low-risk population. From the initial 2,634 observational cases, 101 participants were excluded due to the following conditions: syphilis (n = 6), immune rheumatic diseases (n = 10), diabetes mellitus and hypertension (n = 18), chronic kidney, liver, or heart diseases (n = 22), and thyroid diseases (n = 45). Consequently, a total of 2533 eligible pregnant women were included in the final analysis (Fig. S1). Notably, none of these women reported the consumption of illicit drugs, alcohol use, or smoking during their current pregnancies. Maternal and neonatal data were systematically collected from the hospital's electronic medical record systems. From the hospitalization information system, we extracted comprehensive data including gravidity, parity, gestational age at delivery, anthropometric measurements (height and weight at delivery), pregnancy complications, pre-pregnancy medical history, substance use history, delivery mode, neonatal sex, and birth parameters (height and weight). Additionally, prenatal screening data were obtained from the specialized screening system, which included maternal age, gestational age and weight at the time of serum testing, along with the corresponding biochemical marker results (fβ-hCG, AFP, and uE3). Biomarker quantification was performed using an automated time-resolved fluorescence immunoassay assay (Wallac 1235, PerkinElmer, Finland) with standardized commercial kits. Specifically, AFP and fβ-hCG levels were measured using kit B067-101Z, while uE3 concentrations were determined using kit B083-301Z (both from PerkinElmer, Finland). The assay demonstrated excellent precision, with inter-assay and intra-assay variability coefficients of less than 2% for AFP, below 3% for fβ-hCG, and under 5% for uE3.

The study protocol received ethical approval from the Institutional Review Board of Changzhou MCHC Hospital (Approval No. ZD201803). In accordance with institutional guidelines and ethical standards, the requirement for written informed consent was waived since the study exclusively utilized retrospectively collected, de-identified data from the hospital's electronic medical records system.

### AFGO definition

In this study, AFGO were defined as the presence of either SGA or LGA infants, as well as LBW or Mac. Neonatal growth classification was determined using gestational age-specific birth weight percentiles based on regional standards for Changzhou City, China [28]. Specifically, SGA was defined as birth weight below the 10th percentile for gestational age, while LGA was classified as birth weight above the 90th percentile. Appropriate-for-gestational-age (AGA) infants were those with birth weights between the 10th and 90th percentiles, inclusive. Additionally, neonates were categorized into three birth weight groups: LBW, (< 2500 g), normal birth weight (NBW, 2500–4000 g), and Mac (> 4000 g) [29].

### Statistical analysis

Descriptive analyses were performed to characterize the demographic profiles of both mothers and newborns. Continuous variables were reported as mean values with standard deviations (SD), while categorical variables were presented as number (%). For group comparisons, the analysis of variance (ANOVA) test was employed for normally distributed continuous variables, whereas the Kruskal–Wallis test was utilized for continuous variables with skewed distributions. Categorical variables were compared using the Chi-square test. Associations between maternal serum markers levels, expressed as multiples of the median (MoM), and gestational duration as well as fetal growth parameters (gestational age, birth length, and weight) were evaluated using general linear models. We applied logistic regression models to calculate odds ratios (OR) along with their corresponding 95% confidence intervals (CI) for AFGO. Non-linear relationships between serum biomarkers and AFGO were explored by fitting smooth curves. Covariates adjusted in the analyses included maternal age, body mass index (BMI) at delivery, gestational weight gain (GWG), gravidity, parity, use of assisted reproductive technology (ART), pregnancy complications, neonatal sex, and serum analytes (excluding the analyte of interest). Additionally, receiver operating characteristic (ROC) curve analysis was conducted to determine the area under the curve (AUC) and assess the predictive performance of maternal parameters during prenatal screening and serum markers in identifying AFGO pregnancies. To enhance the prediction of AFGO, we constructed four machine learning-based models: Gradient Boosting Machine (GBM), which sequentially builds decision trees to iteratively correct errors, demonstrating strong performance on structured data but requiring careful parameter tuning to avoid overfitting; Generalized Linear Model (GLM), which assumes linear relationships between inputs and outcomes, offering simplicity and interpretability as a baseline model but struggling with nonlinear patterns; Random Forest (RF), which constructs multiple decision trees using random subsets of data and features, effectively handling noisy datasets while providing feature importance rankings; and Deep Learning (DL), which utilizes layered neural networks to automatically learn hierarchical representations, excelling at capturing intricate patterns but demanding substantial computational resources and large datasets. These models were selected for their complementary strengths, with GLM prioritizing simplicity and interpretability, GBM and RF balancing predictive power with practicality, and DL offering maximum flexibility for complex problems. Their implementation was constrained by the Empower Stats software (version 4.1) used in this study, which supported only these four models as default analytical tools. The modeling incorporated eight maternal parameters and three serum markers from prenatal screening, including age, height, weight, BMI, gravidity, parity, ART, gestational age, AFP, fβ-hCG, and uE3. Data collected from participants in the study cohort were randomly split into a 7:3 ratio. The larger subset (70%) was utilized for model training, while the smaller subset (30%) served as the test set. Model performance was assessed using metrics such as the AUC, specificity, sensitivity, positive predictive value (PPV), and negative predictive value (NPV).

## Results

### Study population characteristics

Tables 1 and 2 present demographic and clinical characteristics of participants stratified by fetal growth outcomes (SGA, AGA, LGA) and birthweight categories (LBW, NBW, Mac). Overall, the mean age (standard deviation, SD) of mothers upon entry into the prenatal screening cohort was 27.8 years (3.17), with 65.69% of the mothers being primiparous. Of the 2533 singleton live births, 211 (8.33%) were classified as SGA, and 373 (14.73%) as LGA. The mean (SD) birthweight was 3389.65 g (467.75), with 3.04% (77) being LBW and 8.09% (205) being Mac. Maternal height, weight/BMI (at prenatal screening and delivery), GWG, as well as GDM prevalence, all increase progressively from SGA to LGA or across fetal birth weight categories (*P* < 0.001 for most comparisons). Cesarean section rates were also higher in groups with LGA/Mac. However, Table 2 highlighted a stronger association between LBW and PTB, with 74.03% of LBW cases being PTB, compared to only 9.95% in SGA (Table 1). Regarding serum analytes measured as MoM, uE3 showed a consistent pattern: it increased from SGA to LGA or across fetal birthweight categories, with significant differences across groups (*P* < 0.001). Specifically, uE3 levels were lowest in SGA/LBW groups and highest in LGA/Mac groups. In contrast, fβ-hCG and AFP did not show significant variations across these groups.

**Table 1:** Descriptive statistics of demographic characteristics and covariates among participants according to fetal growth outcomes (n = 2533)

Characteristics	SGA (n = 211)	AGA (n = 1949)	LGA (n = 373)	*P* value
Age (years)	27.25 ± 3.20	27.73 ± 3.14	28.49 ± 3.25	< 0.001
Height (cm)	160.13 ± 4.19	161.66 ± 4.65	162.89 ± 4.79	0.053
Weight at prenatal screening (kg)	54.28 ± 7.88	58.07 ± 8.83	62.85 ± 9.35	< 0.001
Weight at delivery (kg)	66.59 ± 8.81	71.22 ± 9.25	77.03 ± 9.75	< 0.001
Gestational weight gain (kg) ^a^	12.31 ± 4.79	13.14 ± 4.68	14.19 ± 5.10	< 0.001
BMI at prenatal screening (kg/m^2^)	21.15 ± 2.83	22.21 ± 3.15	23.68 ± 3.35	< 0.001
BMI at delivery (kg/m^2^)	25.95 ± 3.18	27.23 ± 3.23	29.02 ± 3.35	< 0.001
Gravidity (times)	1.73 ± 0.99	1.85 ± 1.06	2.26 ± 1.22	< 0.001
Primipara	156 (73.93%)	1320 (67.73%)	188 (50.40%)	< 0.001
Assisted reproduction	2 (0.95%)	53 (2.72%)	8 (2.14%)	0.263
Gestational age at prenatal screening (day)	121.80 ± 5.68	122.43 ± 6.26	122.23 ± 6.10	0.416
Gestational age at delivery (week)	38.94 ± 2.02	39.01 ± 1.40	38.63 ± 1.36	< 0.001
Systolic BP at delivery (mmHg)	120.90 ± 13.85	120.55 ± 11.82	119.69 ± 12.16	0.322
Diastolic BP at delivery (mmHg)	75.14 ± 10.06	74.08 ± 8.35	73.09 ± 8.25	0.014
GDM	9 (4.27%)	132 (6.77%)	49 (13.14%)	< 0.001
ICP	103 (37.7)	99 (35.5)	82 (29.4)	0.101
PE	18 (8.53%)	74 (3.80%)	13 (3.49%)	0.004
PIH	2 (0.95%)	44 (2.26%)	5 (1.34%)	0.265
PTB	21 (9.95%)	62 (3.18%)	16 (4.29%)	< 0.001
Cesarean section	48 (62.34%)	1033 (45.89%)	132 (64.39%)	< 0.001
Neonatal sex (male)	43 (55.84%)	1140 (50.64%)	136 (66.34%)	< 0.001
Neonatal height (cm)	45.49 ± 3.38	49.98 ± 0.40	51.28 ± 1.39	< 0.001
Neonatal weight (gram)	2089.22 ± 332.89	3353.66 ± 327.61	4273.27 ± 230.79	< 0.001
Serum analytes (MoM)				
fβ-hCG	1.34 ± 1.10	1.12 ± 0.77	1.24 ± 0.98	0.118
AFP	1.25 ± 1.20	1.01 ± 0.34	1.04 ± 0.36	0.130
uE3	0.94 ± 0.25	1.06 ± 0.31	1.10 ± 0.31	< 0.001

Variables were presented as mean ± SD and frequency (%) ^a^ From prenatal screening for Down's syndrome to delivery

*SGA/AGA/LGA* small/appropriate/large for gestational age, *BMI* body mass index, *BP* blood pressure, *GDM* gestational diabetes mellitus, *ICP* intrahepatic cholestasis of pregnancy, *PE* Preeclampsia, *PIH* pregnancy induced hypertension, *PTB* preterm birth, *MoM* multiple of the median, *fβ-hCG* free β-human chorionic gonadotropin, *AFP* α-fetoprotein, *uE3* unconjugated estriol, *SD* standard deviation

**Table 2:** Descriptive statistics of demographic characteristics and covariates among participants according to fetal birth weight (n = 2533)

Characteristics	LBW (n = 77)	NBW (n = 2251)	Mac (n = 205)	*P* value
Age (years)	27.68 ± 3.38	27.78 ± 3.16	28.07 ± 3.28	0.289
Height (cm)	160.77 ± 4.82	161.59 ± 4.64	163.41 ± 4.64	< 0.001
Weight at prenatal screening (kg)	57.71 ± 9.73	58.08 ± 8.90	62.94 ± 9.57	< 0.001
Weight at delivery (kg)	68.00 ± 9.89	71.23 ± 9.38	78.00 ± 9.90	< 0.001
Gestational weight gain (kg) ^a^	10.38 ± 4.96	13.15 ± 4.64	15.06 ± 5.40	< 0.001
BMI at prenatal screening (kg/m^2^)	22.29 ± 3.35	22.23 ± 3.19	23.54 ± 3.29	< 0.001
BMI at delivery (kg/m^2^)	26.30 ± 3.42	27.26 ± 3.28	29.18 ± 3.26	< 0.001
Gravidity (times)	1.97 ± 1.17	1.88 ± 1.08	2.10 ± 1.16	0.022
Primipara	54 (70.13%)	1492 (66.28%)	118 (57.56%)	0.03
Assisted reproduction	1 (1.30%)	60 (2.67%)	2 (0.98%)	0.263
Gestational age at prenatal screening (day)	123.48 ± 6.35	122.37 ± 6.22	121.64 ± 5.71	0.083
Gestational age at delivery (week)	34.64 ± 2.58	39.05 ± 1.19	39.42 ± 1.01	< 0.001
Systolic BP at delivery (mmHg)	128.83 ± 19.56	120.20 ± 11.63	120.04 ± 11.84	< 0.001
Diastolic BP at delivery (mmHg)	78.36 ± 12.58	73.97 ± 8.33	72.98 ± 7.96	0.002
GDM	1 (1.30%)	162 (7.20%)	27 (13.17%)	< 0.001
ICP	7 (9.09%)	114 (5.06%)	11 (5.37%)	0.293
PE	17 (22.08%)	83 (3.69%)	5 (2.44%)	< 0.001
PIH	2 (2.60%)	44 (1.95%)	5 (2.44%)	0.835
PTB	57 (74.03%)	42 (1.87%)	0 (0.00%)	< 0.001
Cesarean section	78 (36.97%)	889 (45.61%)	246 (65.95%)	< 0.001
Neonatal sex (male)	78 (36.97%)	998 (51.21%)	243 (65.15%)	< 0.001
Neonatal height (cm)	49.02 ± 2.30	49.91 ± 0.86	50.65 ± 1.27	< 0.001
Neonatal weight (gram)	2679.34 ± 370.74	3346.10 ± 333.48	4019.01 ± 368.68	< 0.001
Serum analytes (MoM)				
fβ-hCG	1.16 ± 0.84	1.13 ± 0.76	1.18 ± 1.00	0.992
AFP	1.07 ± 0.77	1.01 ± 0.34	1.04 ± 0.34	0.071
uE3	0.94 ± 0.25	1.06 ± 0.31	1.10 ± 0.30	< 0.001

Variables were presented as mean ± SD and frequency (%)

*LBW/NBW* low/normal birth weight, *Mac* macrosomia, *BMI* body mass index, *BP* blood pressure, *GDM* gestational diabetes mellitus, *ICP* intrahepatic cholestasis of pregnancy, *PE* Preeclampsia, *PIH* pregnancy induced hypertension, *PTB* preterm birth, *MoM* multiple of the median, *fβ-hCG* free β-human chorionic gonadotropin, *AFP* α-fetoprotein, *uE3* unconjugated estriol, *SD* standard deviation

^a^From prenatal screening for Down's syndrome to delivery

### Maternal serum analytes and fetal growth

Table 3 shows regression coefficients for fetal growth associated with MoM values of maternal serum analytes. In unadjusted models, fβ-hCG showed no significant association with gestational age, birth length, or birth weight (*P* > 0.05), whereas AFP and uE3 were significantly correlated with all three fetal growth indicators (*P* < 0.05). After adjusting for maternal age, BMI at delivery, gestational weight gain, gravidity, parity, use of assisted reproductive technology, pregnancy complications, neonatal sex, and serum analytes (excluding the analyte being treated as the target), a one standard deviation (SD) increase in uE3 MoM was associated with increased birth length (β = 0.11 cm, 95% CI: 0.07 to 0.15) and weight (β = 63.79 g, 95% CI: 48.83 to 78.76). Conversely, a one SD increase in AFP MoM was associated with reduced birth length (β = -0.06 cm, 95% CI: −0.11 to − 0.02) and weight (β = − 13.28 g, 95% CI: − 28.69 to 2.14). Additionally, a one SD increase in fβ-hCG MoM was associated with increased gestational age (β = 0.06 weeks, 95% CI: 0 to 0.11) and weight (β = 24.5 g, 95% CI: 9.48 to 39.53).

**Table 3:** Regression coefficients [β (95% CI)] for fetal growth indices associated with serum analytes for prenatal screening

Serum analytes (MoM)	Gestational age (weeks)		Birth length (cm)^e^		Birth weight (g)^e^	
	β (95%CI)	*P*	β (95%CI)	*P*	β (95%CI)	*P*
Unadjusted						
fβ-hCG (continuous)	0.06 (− 0.01, 0.13)	0.110	0.00 (− 0.05, 0.06)	0.906	21.88 (− 0.76, 44.52)	0.058
fβ-hCG (per-SD increase)	0.05 (− 0.01, 0.10)	0.110	0.00 (− 0.04, 0.05)	0.906	17.60 (− 0.61, 35.81)	0.058
AFP (continuous)	− 0.53 (− 0.67, − 0.38)	< 0.001	− 0.33 (− 0.45, − 0.21)	< 0.001	− 76.41 (− 122.52, − 30.29)	0.001
AFP (per-SD increase)	− 0.21 (− 0.26, − 0.15)	< 0.001	− 0.13 (− 0.18, − 0.08)	< 0.001	− 30.13 (− 48.32, − 11.95)	0.001
uE3 (continuous)	− 0.23 (− 0.42, − 0.04)	0.016	0.19 (0.04, 0.34)	0.014	139.24 (79.77, 198.71)	< 0.001
uE3 (per-SD increase)	− 0.07 (− 0.13, − 0.01)	0.016	0.06 (0.01, 0.10)	0.014	42.49 (24.34, 60.64)	< 0.001
Adjusted^a^						
fβ-hCG (continuous)^b^	0.07 (0.00, 0.14)	0.042	0.02 (− 0.03, 0.07)	0.405	30.47 (11.79, 49.15)	0.001
fβ-hCG (per-SD increase)^b^	0.06 (0.00, 0.11)	0.042	0.02 (− 0.02, 0.06)	0.405	24.50 (9.48, 39.53)	0.001
AFP (continuous)^c^	− 0.45 (− 0.59, − 0.30)	< 0.001	− 0.16 (− 0.27, − 0.06)	0.002	− 33.67 (− 72.76, 5.43)	0.092
AFP (per-SD increase)^c^	− 0.18 (− 0.23, − 0.12)	< 0.001	− 0.06 (− 0.11, − 0.02)	0.002	− 13.28 (− 28.69, 2.14)	0.092
uE3 (continuous)^d^	− 0.22 (− 0.40, − 0.04)	0.018	0.35 (0.22, 0.48)	< 0.001	209.05 (160.02, 258.07)	< 0.001
uE3 (per-SD increase)^d^	− 0.07 (− 0.12, − 0.01)	0.018	0.11 (0.07, 0.15)	< 0.001	63.79 (48.83, 78.76)	< 0.001

*MoM* multiple of the median, *CI* confidence interval, *fβ-hCG* free β-human chorionic gonadotropin, *SD* standard deviation, *AFP* α-fetoprotein, *uE3* unconjugated estriol, *BMI* body mass index, *BP* blood pressure

^a^Adjusted for maternal age, BMI at delivery, gravidity, parity, asystolic and diastolic BP at delivery, assisted reproduction, pregnancy complications, and neonatal sex

^b^Additionally corrected for AFP and uE3

^c^Additionally corrected for fβ-hCG and uE3

^d^Additionally corrected for fβ-hCG and AFP

^e^Additionally corrected for gestational age

### Maternal serum analytes and AFGO

**Fig. 1:**
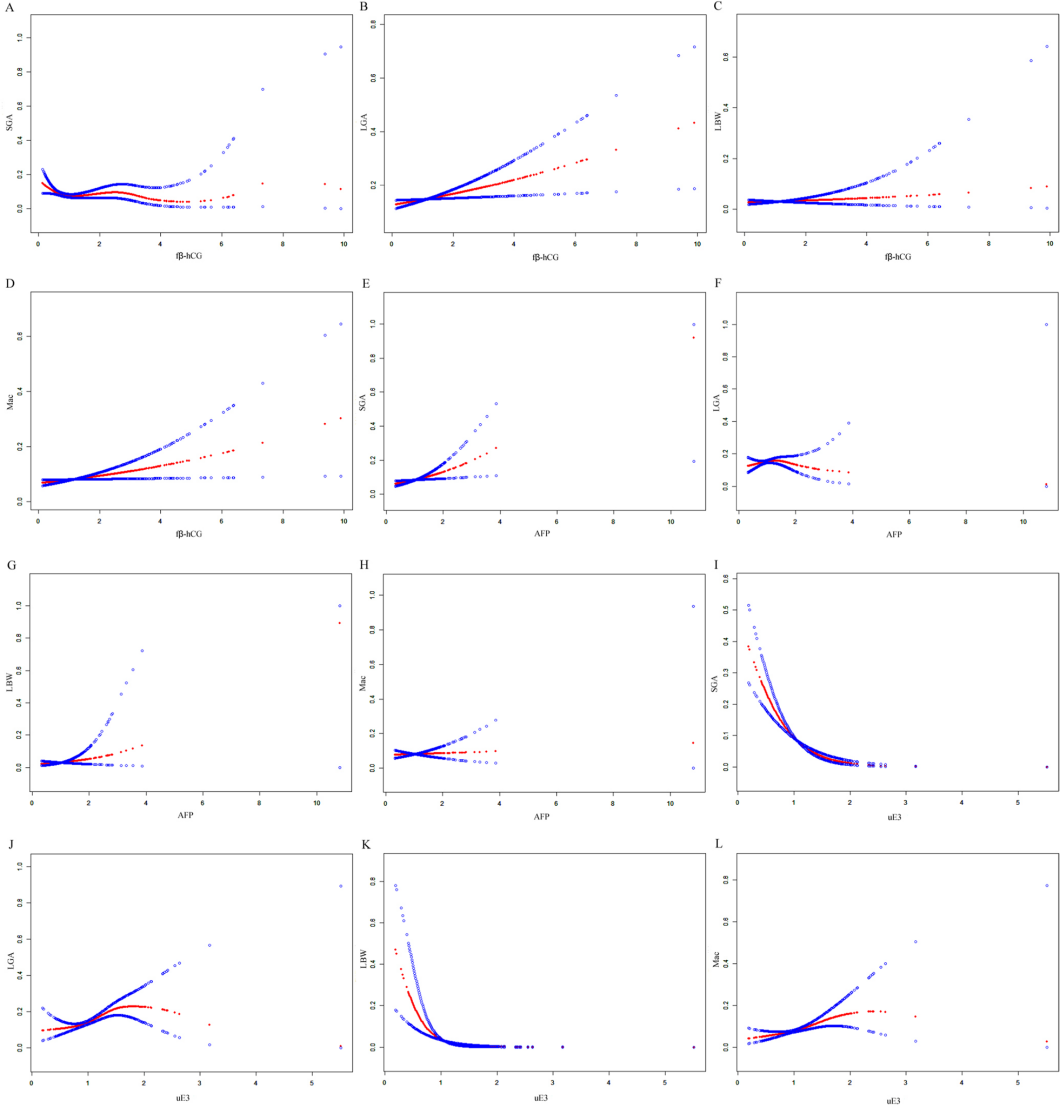
Relationship between maternal serum triple markers and adverse fetal growth outcomes by smooth curve fitting analysis. **A**–**D** fβ-hCG and small-for-gestational age, large-for-gestational age, low birth weight, and macrosomia. **E**–**H** AFP and small-for-gestational age, large-for-gestational age, low birth weight, and macrosomia. **I**–**L** uE3 and small-for-gestational age, large-for-gestational age, low birth weight, and macrosomia. Adjustment factors included maternal age, body mass index at delivery, gestational weight gain, gravidity, parity, use of assisted reproductive technology, pregnancy complications, neonatal sex, and serum analytes (excluding the analyte of interest)

Table 4 presents the ORs and 95% CIs for AFGO associated with serum analyte levels during prenatal screening. In unadjusted models, fβ-hCG showed no significant association with SGA, LGA, LBW, or Mac (all *P* > 0.05). However, after adjustment, fβ-hCG demonstrated a significant positive association with LGA (for per SD increase: OR = 1.15, 95% CI: 1.03–1.28, *P* = 0.015) and Mac (for per SD increase: OR = 1.16, 95% CI: 1.01–1.32, *P* = 0.030). Similarly, AFP did not show significant associations with any growth outcomes in unadjusted models. After adjustment, however, AFP was significantly positively associated with SGA (for per SD increase: OR = 1.21, 95% CI: 1.04–1.42, P = 0.014) but not with other growth outcomes. Notably, uE3 exhibited a strong inverse relationship with SGA (for per SD increase: OR = 0.51, 95% CI: 0.42–0.61, *P* < 0.001) and LBW (for per SD increase: OR = 0.31, 95% CI: 0.19–0.50, *P* < 0.001), as well as a significant positive relationship with LGA (for per SD increase: OR = 1.22, 95% CI: 1.08–1.37, *P* = 0.001) and Mac (for per SD increase: OR = 1.22, 95% CI: 1.06–1.42, *P* = 0.006) in adjusted models. Additionally, smooth curve fitting analysis with multivariable adjustment revealed nonlinear associations between serum analytes and AFGO (Fig. 1).

**Table 4:** ORs and 95% CIs for adverse fetal growth outcomes with serum analytes for prenatal screening

Serum analytes (MoM)	SGA		LGA		LBW		Mac	
	OR (95% CI)	*P* value	OR (95% CI)	*P* value	OR (95% CI)	*P* value	OR (95% CI)	*P* value
Unadjusted								
fβ-hCG (continuous)	1.04 (0.87, 1.23)	0.686	1.08 (0.95, 1.23)	0.241	1.27 (1.03, 1.56)	0.025	1.16 (1.00, 1.35)	0.057
fβ-hCG (per-SD increase)	1.03 (0.90, 1.18)	0.686	1.06 (0.96, 1.18)	0.241	1.21 (1.02, 1.43)	0.025	1.13 (1.00, 1.27)	0.057
AFP (continuous)	1.29 (0.97, 1.70)	0.075	1.17 (0.91, 1.50)	0.217	2.16 (1.30, 3.57)	0.003	1.10 (0.80, 1.52)	0.543
AFP (per-SD increase)	1.11 (0.99, 1.23)	0.075	1.06 (0.96, 1.17)	0.217	1.35 (1.11, 1.65)	0.003	1.04 (0.92, 1.18)	0.543
uE3 (continuous)	0.17 (0.09, 0.29)	< 0.001	1.64 (1.17, 2.29)	0.004	0.20 (0.08, 0.50)	< 0.001	1.57 (1.04, 2.38)	0.032
uE3 (per-SD increase)	0.58 (0.48, 0.69)	< 0.001	1.16 (1.05, 1.29)	0.004	0.61 (0.47, 0.81)	< 0.001	1.15 (1.01, 1.30)	0.032
Adjusted^a^								
fβ-hCG (continuous)^b^	0.91 (0.75, 1.11)	0.342	1.18 (1.03, 1.36)	0.015	1.14 (0.82, 1.59)	0.445	1.20 (1.02, 1.41)	0.030
fβ-hCG (per-SD increase)^b^	0.93 (0.79, 1.08)	0.342	1.15 (1.03, 1.28)	0.015	1.11 (0.85, 1.45)	0.445	1.16 (1.01, 1.32)	0.030
AFP (continuous)^c^	1.64 (1.10, 2.43)	0.014	0.98 (0.73, 1.32)	0.917	1.77 (0.66, 4.71)	0.255	1.07 (0.68, 1.67)	0.770
AFP (per-SD increase)^c^	1.21 (1.04, 1.42)	0.014	0.99 (0.88, 1.12)	0.917	1.25 (0.85, 1.84)	0.255	1.03 (0.86, 1.22)	0.770
uE3 (continuous)^d^	0.11 (0.06, 0.20)	< 0.001	1.90 (1.29, 2.80)	0.001	0.02 (0.00, 0.10)	< 0.001	1.94 (1.21, 3.12)	0.006
uE3 (per-SD increase)^d^	0.51 (0.42, 0.61)	< 0.001	1.22 (1.08, 1.37)	0.001	0.31 (0.19, 0.50)	< 0.001	1.22 (1.06, 1.42)	0.006

*SGA/LGA* small/large for gestational age, *LBW* low birth weight, *Mac* macrosomia, *OR* odds ratio, *CI* confidence interval, *fβ-hCG* free β-human chorionic gonadotropin, *SD* standard deviation, *AFP* α-fetoprotein, *uE3* unconjugated estriol, *BMI* body mass index, *BP* blood pressure

^a^Adjusted for maternal age, BMI at delivery, gravidity, parity, asystolic and diastolic BP at delivery, assisted reproduction, pregnancy complications, gestational age, and neonatal sex

^b^Additionally corrected for AFP and uE3. ^c^ Additionally corrected for fβ-hCG and uE3. ^d^ Additionally corrected for fβ-hCG and AFP

### Predicting AFGO with serum analytes and related models

**Fig. 2:**
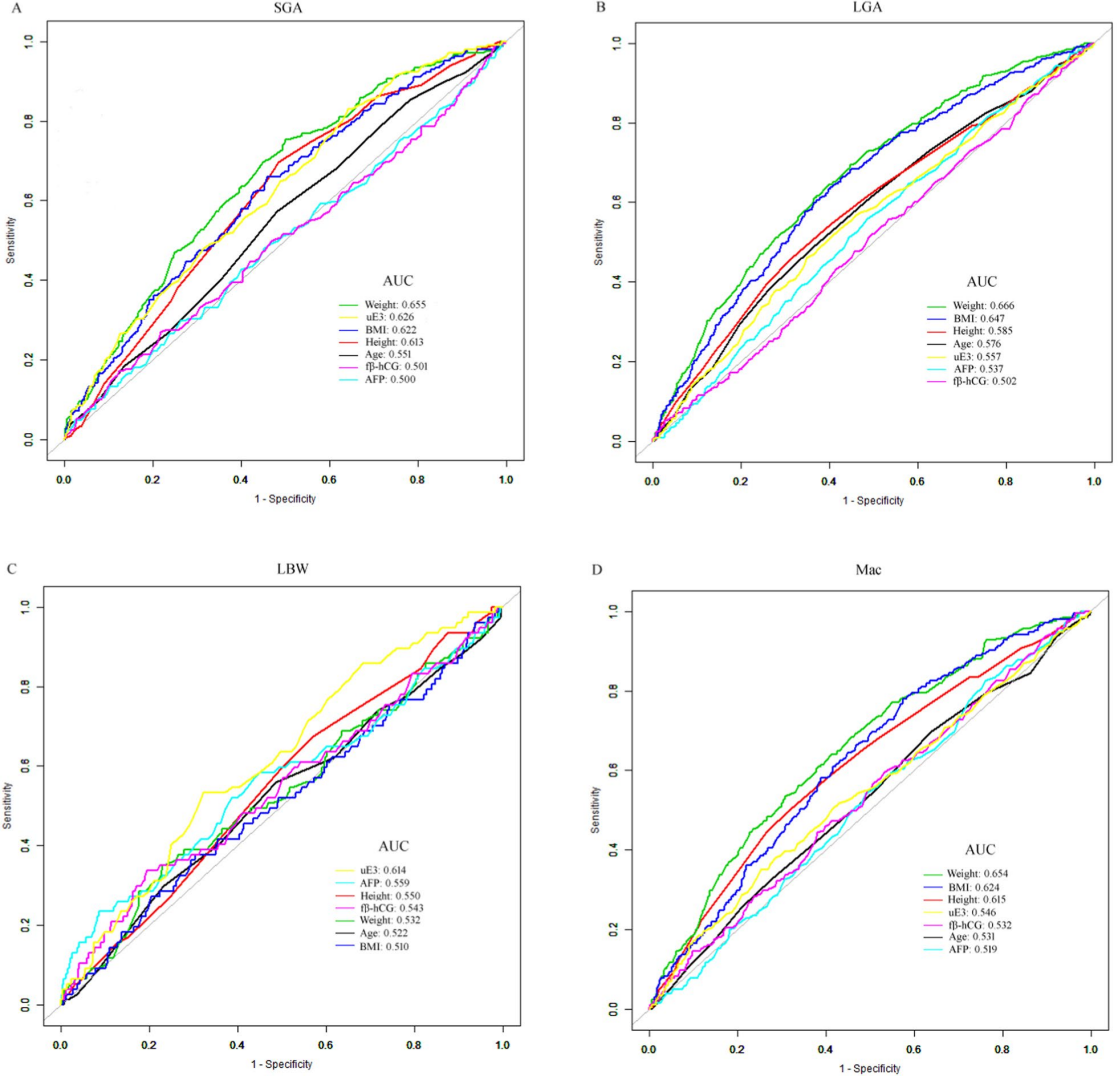
Comparison of the potential of various variables in predicting adverse fetal growth outcomes by the receiver operating characteristic (ROC) curve analysis. **A** Small-for-gestational age. **B** Large-for-gestational age. **C** Low birth weight. **D** Macrosomia. ROC curves showed the ability of serum uE3 to predict adverse fetal growth outcomes was superior to that of either fβ-hCG or AFP alone

**Fig. 3:**
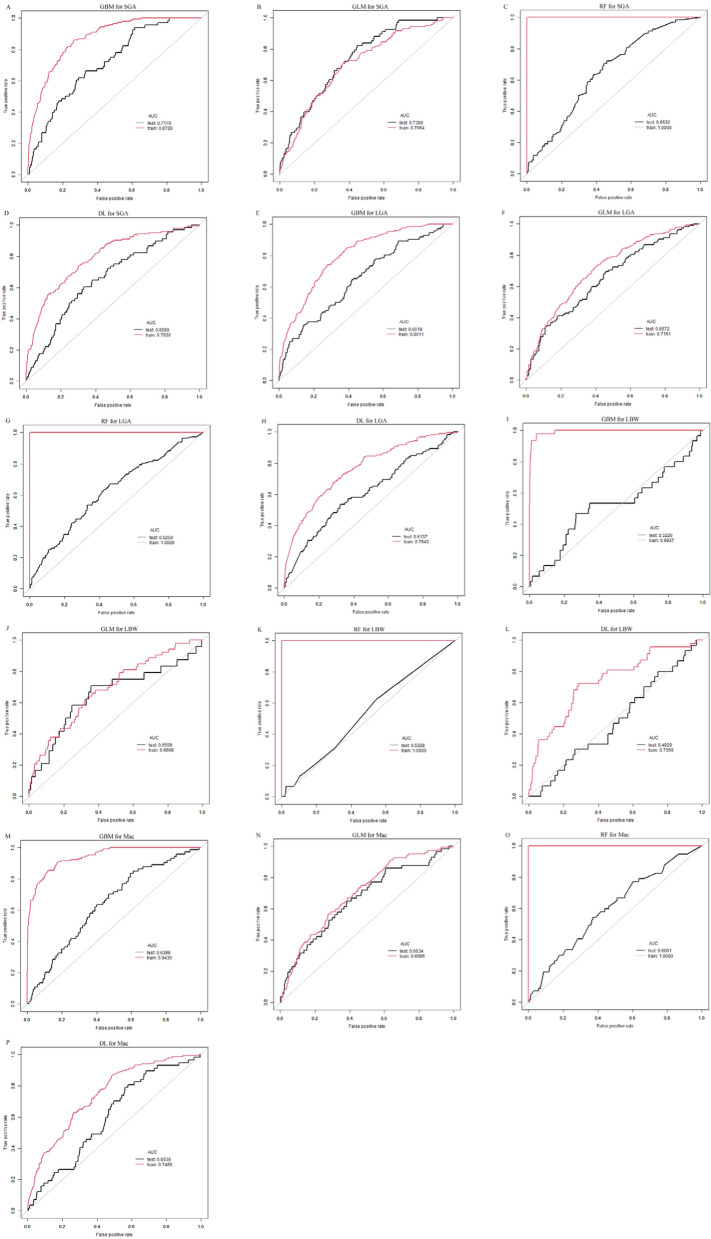
Performance of four machine learning models for predicting adverse fetal growth outcomes by ROC curve analysis in training and validation sets. **A**–**D** Four machine learning-based prediction models for small-for-gestational age. **E**–**H** Four machine learning-based prediction models for large-for-gestational age. **I**–**L** Four machine learning-based prediction models for low birth weight. **M**–**P** Four machine learning-based prediction models for macrosomia. The most significant models for identifying small-for-gestational age were Gradient Boosting Machine and Generalized Linear Model, with an AUC of 0.873 and 0.706 in the training set (n = 1782, 143 SGA), and an AUC of 0.717 and 0.739 in the test set (n = 751, 68 SGA), respectively

ROC curves were generated to assess the capability of serum analytes and clinical parameters, individually, to predict AFGO (Fig. 2). The ability of serum uE3 to predict AFGO was superior to that of either fβ-hCG or AFP alone, as evidenced by the AUC values from ROC analyses (for SGA: 0.626 vs. 0.501/0.500; for LGA: 0.557 vs. 0.502/0.537; for LBW: 0.614 vs. 0.543/0.559; for Mac: 0.546 vs. 0.532/0.519; Table 5). Since integrating multiple biomarkers is more effective than using a single biomarker for identifying AFGO, we aimed to evaluate the diagnostic performance of combined prenatal screening variables by developing machine learning models. Four models (GBM, GLM, RF, and DL) were built (Fig. 3). The predictive performance for AFGO across different models is presented in Table 6. GBM and GLM demonstrated varying performance in identifying SGA neonates, with GBM achieving higher discriminatory power (AUC = 0.873) in the training set (n = 1782, 143 SGA cases) compared to GLM (AUC = 0.706), though GBM’s performance declined in the test set (AUC = 0.717 vs. GLM’s 0.739), suggesting potential overfitting. Clinically, GBM’s optimal threshold (0.15) prioritized specificity (92.50% training, 90.78% test) over sensitivity (51.75% training, 26.47% test), making it suitable for ruling out SGA (high NPV: 95.65% training, 92.54% test) but less effective for screening due to low sensitivity. Conversely, GLM’s threshold (0.12) balanced specificity (85.65% training, 84.50% test) and sensitivity (39.71% training, 40.56% test) but yielded lower PPV (21.60% training, 18.59% test).

**Table 5:** Accuracy of demographic parameters and serum analytes at prenatal screening to predict adverse growth outcomes

Variables	AUC	95% CI	Best threshold	Specificity (%)	Sensitivity (%)	PPV (%)	NPV (%)
SGA							
Weight at prenatal screening (kg)	0.655	0.618, 0.692	57.73	49.91	75.36	12.03	95.71
uE3 (MoM)	0.626	0.588, 0.664	1.14	35.79	82.94	10.50	95.85
BMI at prenatal screening (kg/m^2^)	0.622	0.583, 0.661	21.66	54.18	65.88	11.56	94.58
Height (cm)	0.613	0.576, 0.651	161.5	51.51	69.67	11.56	94.92
Age (years)	0.551	0.511, 0.590	27.5	51.94	57.35	9.78	93.06
fβ-hCG (MoM)	0.501	0.458, 0.544	0.62	78.17	27.01	10.11	92.18
AFP (MoM)	0.500	0.458, 0.543	1.18	73.64	29.86	9.33	92.03
LGA							
Weight at prenatal screening (kg)	0.666	0.637, 0.695	58.95	60.56	64.08	21.91	90.71
BMI at prenatal screening (kg/m^2^)	0.647	0.617, 0.676	22.30	59.73	63.81	21.50	90.52
Height (cm)	0.585	0.553, 0.617	163.5	68.63	45.84	20.17	88.00
Age (years)	0.576	0.545, 0.608	28.5	63.89	48.53	18.83	87.79
uE3 (MoM)	0.557	0.525, 0.589	1.12	65.05	46.11	18.55	87.48
AFP (MoM)	0.537	0.506, 0.568	0.97	51.57	55.76	16.59	87.10
fβ-hCG (MoM)	0.502	0.470, 0.534	0.95	51.25	51.21	15.35	85.88
LBW							
uE3 (MoM)	0.614	0.552, 0.675	0.91	67.83	53.25	4.93	97.88
AFP (MoM)	0.559	0.485, 0.633	1.48	91.29	23.38	7.76	97.44
Height (cm)	0.550	0.487, 0.613	162.3	42.91	67.53	3.58	97.68
fβ-hCG (MoM)	0.543	0.472, 0.615	1.53	80.62	33.77	5.18	97.49
Weight at prenatal screening (kg)	0.532	0.462, 0.602	51.8	76.95	35.06	4.55	97.42
Age (years)	0.522	0.454, 0.591	27.5	51.38	55.84	3.48	97.38
BMI at prenatal screening (kg/m^2^)	0.510	0.440, 0.579	20.44	69.64	37.66	3.75	97.27
Mac							
Weight at prenatal screening (kg)	0.654	0.615, 0.692	61.05	69.16	53.66	13.29	94.43
BMI at prenatal screening (kg/m^2^)	0.624	0.587, 0.661	21.15	42.43	78.05	10.67	95.64
Height (cm)	0.615	0.574, 0.655	163.5	67.97	50.24	12.15	93.94
uE3 (MoM)	0.546	0.503, 0.589	1.22	74.66	35.12	10.88	92.89
fβ-hCG (MoM)	0.532	0.491, 0.573	1.09	61.94	44.39	9.31	92.67
Age (years)	0.531	0.489, 0.572	26.5	36.34	69.76	8.80	93.17
AFP (MoM)	0.519	0.478, 0.559	0.77	23.88	82.44	8.71	93.92

*AUC* area under the curve, *CI* confidence interval, *PPV* positive predictive value, *NPV* negative predictive value, *SGA/LGA* small/large for gestational age, *LBW* low birth weight, *Mac* macrosomia, *uE3* unconjugated estriol, *MoM* multiple of the median, *BMI* body mass index, *fβ-hCG* free β-human chorionic gonadotropin, *AFP* α-fetoprotein

**Table 6:** The performance of different models in discriminating adverse growth outcomes from the cohort

Parameters	Training set (n = 1782, SGA 143, LGA 261, LBW 47, Mac 131)							Test set (n = 751, SGA 68, LGA 112, LBW 30, Mac 74)						
	AUC	95% CI	Best threshold	Specificity (%)	Sensitivity (%)	PPV (%)	NPV (%)	AUC	95% CI	Best threshold	Specificity (%)	Sensitivity (%)	PPV (%)	NPV (%)
SGA														
GBM	0.873	0.835, 0.911	0.15	92.50	51.75	37.56	95.65	0.717	0.646, 0.788	0.15	90.78	26.47	22.22	92.54
GLM	0.706	0.657, 0.755	0.12	85.65	39.71	21.60	93.45	0.739	0.669, 0.809	0.12	84.50	40.56	18.59	94.22
RF	1.000	0.979, 1.000	0.10	89.93	100	46.43	100	0.653	0.580, 0.726	0.10	66.47	51.47	13.26	93.22
DL	0.793	0.748, 0.838	0.13	90.67	47.55	30.77	95.20	0.659	0.586, 0.732	0.13	88.58	22.06	16.13	91.95
LGA														
GBM	0.801	0.768, 0.834	0.17	74.75	70.11	32.28	93.58	0.652	0.593, 0.711	0.17	67.76	49.11	21.07	88.37
GLM	0.716	0.679, 0.753	0.19	80.74	47.89	29.9	90.03	0.657	0.599, 0.715	0.19	78.4	41.96	25.41	88.52
RF	1.000	0.989, 1.000	0.24	98.29	100	90.94	100	0.625	0.566, 0.684	0.24	75.74	41.96	23.27	88.16
DL	0.764	0.729, 0.799	0.33	87.25	47.51	38.99	90.64	0.614	0.555, 0.673	0.33	82.47	33.04	24.83	87.54
LBW														
GBM	0.994	0.978, 1.000	0.22	99.65	80.85	86.36	99.48	0.523	0.416, 0.630	0.22	97.64	6.67	96.17	10.53
GLM	0.690	0.605, 0.775	0.08	96.86	18.87	15.62	97.50	0.651	0.542, 0.760	0.08	97.53	12.50	14.29	97.13
RF	1.000	0.936, 1.000	0.18	99.60	100	87.04	100	0.533	0.426, 0.640	0.16	96.95	6.67	8.33	96.15
DL	0.736	0.653, 0.819	0.09	94.47	34.04	14.29	98.14	0.483	0.379, 0.587	0.09	94.87	0	0	95.80
Mac														
GBM	0.943	0.915, 0.971	0.15	98.06	64.89	72.65	97.24	0.64	0.569, 0.711	0.15	95.43	10.81	20.51	90.74
GLM	0.699	0.648, 0.750	0.12	87.21	36.49	20.53	93.81	0.663	0.593, 0.733	0.12	87.46	31.58	17.14	93.96
RF	1.000	0.977, 1.000	0.04	65.54	100	20.82	100	0.6	0.529, 0.671	0.15	36.17	78.95	9.22	95.44
DL	0.747	0.697, 0.797	0.47	91	36.49	26.87	94.05	0.604	0.533, 0.675	0.47	86.89	19.30	10.78	92.91

*SGA/LGA* small/large for gestational age, *LBW* low birth weight, *Mac* macrosomia, *AUC* area under the curve, *CI* confidence interval, *PPV* positive predictive value, *NPV* negative predictive value, *GBM* gradient boosting machine, *GLM* generalized linear model, *RF* random forest, *DL* deep learning

## Discussion

This study explored the differential roles of maternal serum analytes in fetal growth and the prediction of AFGO. Unadjusted analyses revealed no significant association of fβ-hCG with gestational age or birth parameters, whereas AFP and uE3 exhibited significant correlations. After adjustments, increases in uE3 MoM were linked to higher birth length and weight, while increases in AFP MoM were inversely related. Notably, uE3 demonstrated a protective effect against SGA and LBW but was positively associated with LGA and Mac. Additionally, uE3 outperformed other markers in predicting AFGO, as indicated by ROC analysis. Machine learning models integrating multiple biomarkers improved diagnostic performance, especially for SGA identification, with GBM and GLM achieving high AUC in both training and test sets. These findings highlight uE3's critical role in fetal growth assessment and suggest the potential of integrating multiple biomarkers and advanced modeling techniques for improved prediction of SGA.

The placenta serves as the primary regulatory organ for maternal serum concentrations of triple screening analytes, directly secreting fβ-hCG and uE3, while precisely modulating the transplacental transfer of AFP. Disruption of these essential placental functions due to pathological conditions can lead to both characteristic alterations in biomarker profiles and subsequent impairment of fetal growth trajectories [30]. However, the association between maternal serum triple makers and AFGO has shown some discrepancies. Most studies have demonstrated a significant inverse correlation between fetal birth weight and maternal serum AFP levels during mid-pregnancy. Notably, both elevated and reduced AFP concentrations are associated with abnormal fetal growth: higher AFP levels correlate with an increased risk of FGR or LBW newborns, while lower levels are linked to LGA infants or macrosomia [5, 31, 32]. Conversely, mid-pregnancy levels of fβ-hCG and uE3 exhibit a positive association with fetal birth weight. Similarly, deviations in these biomarkers, either elevated or reduced, also serve as potential risk factors for abnormal growth outcomes. Specifically, elevated fβ-hCG and uE3 levels are associated with LGA infants or macrosomia, whereas decreased levels correlate with FGR or LBW newborns [4, 6, 7, 32, 33]. In this study, we confirmed nonlinear associations between the serum triple markers (AFP, fβ-hCG, and uE3) and AFGO (SGA, LGA, LBW, and Mac). Importantly, the directionality of these associations aligned with previous observations. However, some studies have also indicated that higher fβ-hCG values are correlated with LBW and SGA newborns [34–36]. Additionally, a recent study's findings did not support the association between elevated serum fβ-hCG levels during mid-pregnancy and both macrosomia and LBW infants [37]. These discrepancies may stem from methodological variations in study design (e.g., adjustment protocols for pregnancy complication), population characteristics (e.g., ethnic heterogeneity), or analytical approaches to biomarker quantification.

While prior research has predominantly explored the correlation between serum triple biomarkers and AFGO, relatively few investigations have systematically examined the efficacy of these markers (either alone or in combination) in predicting AFGO with moderate accuracy in cases of SGA [38–40]. To our knowledge, Chen et al. have conducted the sole investigation to date employing ML techniques to develop an SGA prediction model integrating triple biomarkers with maternal characteristics. However, RF algorithm for mid-pregnancy risk stratification demonstrated suboptimal diagnostic performance, achieving an AUC of 0.68, which falls below the clinically acceptable threshold for screening utility [27]. In the current study, we systematically evaluated both individual biomarkers and four developed ML models for AFGO prediction. Regarding single-marker performance, we provide the first clinical evidence that uE3 demonstrates superior predictive capacity for SGA detection, achieving an AUC of 0.63. Notably, GBM and GLM models showed enhanced predictive accuracy during internal validation, with AUCs of 0.72 and 0.74, respectively for SGA prediction. These computational tools potentially extend the clinical application scope of prenatal triple-marker screening, though their translational value necessitates external validation across multiethnic cohorts to ensure population-wide generalizability. In practice, the proposed model could be seamlessly integrated into existing prenatal care workflows as a secondary screening tool following routine second-trimester aneuploidy screening. Using already available biomarker data and maternal demographics, the algorithm could automatically generate individualized SGA risk scores during mid-pregnancy. Pregnant women classified as high-risk could then be referred for intensified monitoring, including serial growth ultrasounds, Doppler studies, and multidisciplinary counseling. This stratified approach may allow for optimized resource allocation by prioritizing high-risk pregnancies for specialized surveillance while reducing unnecessary interventions in low-risk populations. Furthermore, identification of atypical biomarker patterns may prompt earlier investigation of underlying rare genetic or placental disorders, enabling timely molecular testing and personalized management strategies.

### Strengths and limitations

The present study offers several strengths and contributes to the existing body of knowledge on the associations between maternal serum analytes and fetal growth outcomes. Firstly, the study employed a rigorous statistical approach, including adjustment for multiple confounding factors, to elucidate the independent effects of uE3, AFP, and fβ-hCG on fetal growth indicators. This analytical rigor enhanced the reliability and validity of the findings. Secondly, the study incorporated a range of fetal growth outcomes, such as gestational age, birth length, birth weight, SGA, LGA, LBW, and Mac, providing a comprehensive assessment of fetal growth. This comprehensiveness allows for a more nuanced understanding of the relationships between maternal serum triple marker and AFGO. Moreover, the study utilized predictive modeling through machine learning algorithms to assess the predictive performance of serum analytes in detecting AFGO. These advanced statistical techniques contribute to the robustness of the study's findings and offer insights into the potential clinical utility of these analytes. The use of multiple models, such as GBM, GLM, RF, and DL, further strengthens the study by allowing for a comparison of predictive performance across different methodologies.

Despite these strengths, the study is not without limitations. Firstly, being observational in nature, causal relationships cannot be definitively established. While the associations between maternal serum analytes and fetal growth outcomes are statistically significant, the study design does not permit the determination of causality. Secondly, the study population may not be fully representative of all pregnant women, limiting the generalizability of the findings. The cohort was restricted to low-risk pregnancies in a single clinic in China, excluding high-risk populations such as those with maternal pre-gestational comorbidities (e.g., diabetes, hypertension), congenital anomalies, or "high-risk" screening results. This restriction, while methodologically necessary to isolate the predictive value of biochemical markers, inherently limits the applicability of the models to high-risk pregnancies, which are more prevalent in diverse clinical settings. Furthermore, the population demographics (e.g., age, ethnicity, socioeconomic status) and healthcare infrastructure of this specific clinic may not reflect other regions or countries, particularly those with differing prenatal care practices or AFGO prevalence rates. Thirdly, although the study adjusted for multiple confounding factors, residual confounding could still influence the results. For example, this study could not systematically collect key confounding variables, including family history, history of adverse pregnancy outcomes (e.g., GDM, PE, FGR, LBW, and Mac), or genetic information, due to the inherent constraints of retrospective data. Importantly, this limitation may confound the observed associations between serological markers and AFGO. Furthermore, reliance on prenatal screening data may introduce biases, as these data are typically collected for clinical purposes rather than research. Finally, while the study demonstrates the predictive performance of serum analytes, their clinical utility in routine prenatal care remains to be established. Current SGA prediction models (GBM AUC = 0.72; GLM AUC = 0.74) fell below the clinically actionable threshold (AUC ≥ 0.75), but this limitation could be addressed through systematic integration of two critical factors: (1) detailed clinical histories, particularly prior adverse pregnancy outcomes such as FGR or PE, and (2) expanded biomarker panels. Such a combined approach is expected to enhance predictive accuracy beyond the 0.75 AUC benchmark, ultimately improving antenatal risk stratification for clinical decision-making. Future studies are needed to validate these findings in larger, more diverse populations and to assess the potential clinical impact of incorporating these analytes into prenatal care algorithms.

## Conclusion

This study demonstrated that among routinely measured biochemical markers for prenatal Down syndrome screening, serum uE3 exhibited higher predictive performance for AFGO than fβ‑hCG and AFP. The incorporation of these biomarkers, along with maternal characteristics, into ML-based models, particularly GBM and GLM, significantly enhanced the predictive accuracy for SGA infants, with robust performance observed across both training and test datasets. These findings highlight the potential of combining standard prenatal screening biomarkers with advanced ML techniques to improve the early identification of SGA, offering a clinically feasible strategy for risk stratification and intervention. Beyond immediate obstetric applications, this approach might hold transformative potential for certain rare disease detection. For example, early recognition of SGA patterns associated with conditions such as congenital hypothyroidism, Beckwith-Wiedemann syndrome, Edwards syndrome, or Angelman syndrome, all of which can present with atypical growth phenotypes, could facilitate prompt genetic evaluation and personalized management. Future research should focus on validating these models in larger and more diverse populations and exploring the integration of additional biomarkers to further improve predictive performance.

## Supplementary Information

Supplementary Material 1: Figure S1. Flow chart for participant selection. *MCHC* Maternity and Child Health Care.

## Data Availability

The datasets used and/or analyzed during the current study are available from the corresponding author upon reasonable request.

## Abbreviations

AFGO: Adverse fetal growth outcomes
SGA: Small for gestational age
LGA: Large for gestational age
LBW: Low birth weight
Mac: Macrosomia
AFP: α-Fetoprotein
fβ-hCG: Free β-human chorionic gonadotropin
uE3: Unconjugated estriol
