# Supplementary material for: Machine learning-augmented biomarkers in mid-pregnancy Down syndrome screening improve prediction of small-for-gestational-age infants
Source: Orphanet J Rare Dis. 2025 Oct 1;20:496. doi: 10.1186/s13023-025-04027-1 (PMC12487514; doi:10.1186/s13023-025-04027-1)
Supplement: Supplementary file 1 — Supplementary Material 1: Figure S1. Flow chart for participant selection. MCHC Maternity and Child Health Care. [file 13023_2025_4027_MOESM1_ESM.pptx]

## Slide 1
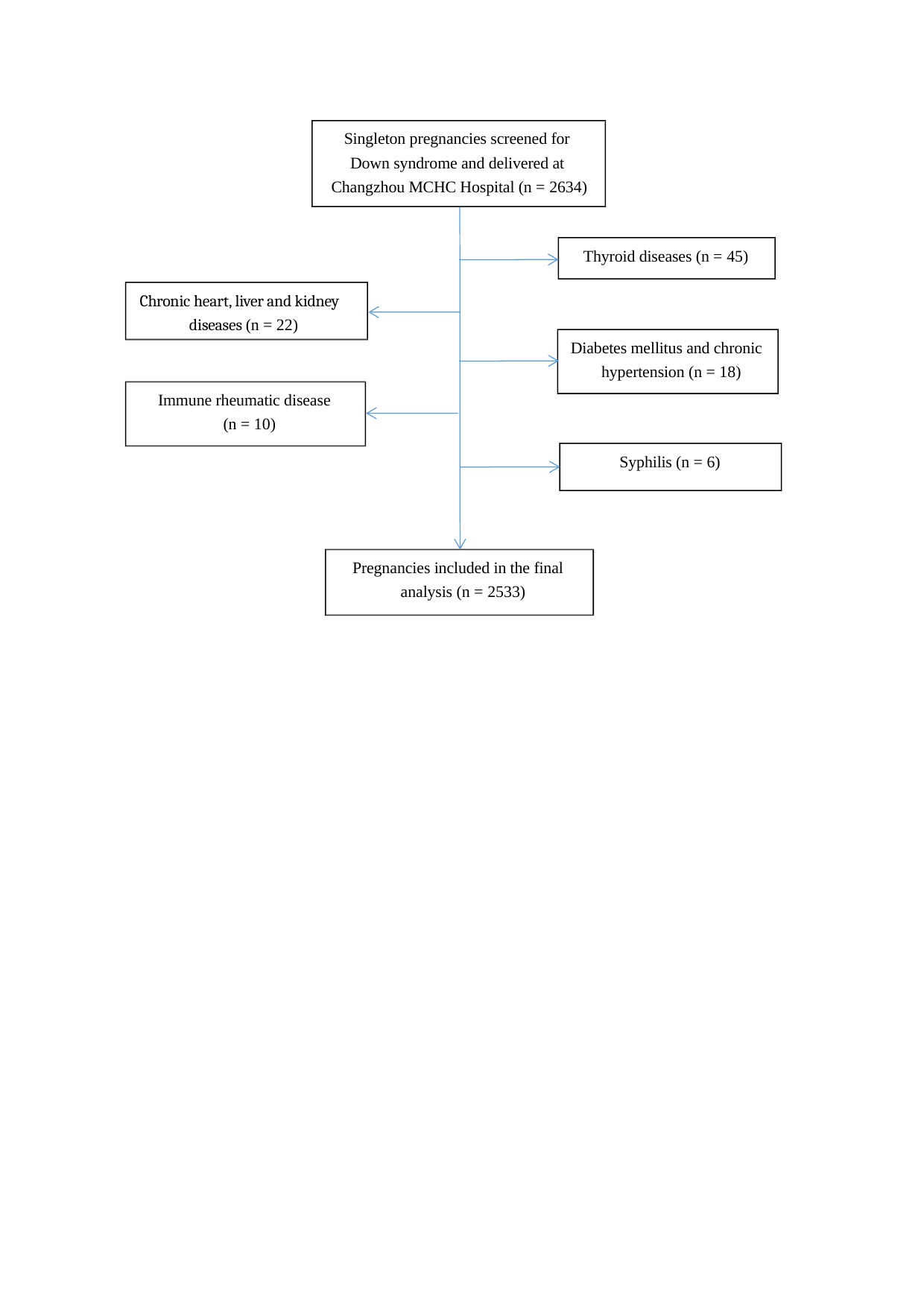

Singleton pregnancies screened for Down syndrome and delivered at Changzhou MCHC Hospital (n = 2634)
Thyroid diseases (n = 45)
Chronic heart, liver and kidney diseases (n = 22)
Diabetes mellitus and chronic hypertension (n = 18)
Immune rheumatic disease (n = 10)
Syphilis (n = 6)
Pregnancies included in the final analysis (n = 2533)
